# Altered thalamus functional connectivity in patients with acute unilateral vestibulopathy: a resting-state fMRI study

**DOI:** 10.3389/fnins.2024.1388213

**Published:** 2024-07-01

**Authors:** Zhengwei Chen, Yaxian Cai, Yueji Liu, Haiyan Liu, Xiu-e Wei, Cunxin Lin, Dan Liu, Lijie Xiao, Liangqun Rong

**Affiliations:** ^1^Department of Neurology, Second Affiliated Hospital of Xuzhou Medical University, Xuzhou, Jiangsu, China; ^2^Department of Neurology, General Hospital of the Yangtze River Shipping, Wuhan, Hubei, China

**Keywords:** acute unilateral vestibulopathy, functional connectivity, vestibular compensation, vestibular neuritis, resting-state fMRI, thalamus

## Abstract

**Objective:**

Acute unilateral vestibulopathy (AUVP) is the second leading cause of peripheral vestibular vertigo. Full recovery of AUVP is related to sufficient central vestibular compensation. It has been confirmed that the vestibular nucleus and vestibular cortex are involved in the process of vestibular compensatory in AUVP patients. However, few studies have focused on the functional compensation of thalamus in patients with AUVP. This study aimed to explore the alterations of resting-state functional connectivity (FC) focused on thalamus using functional magnetic resonance imaging (fMRI) in AUVP patients.

**Methods:**

Data of 3D-T1 and resting-state fMRI were collected from 40 AUVP patients and 35 healthy controls (HC). Seeds-based (bilateral thalamus) FC was analyzed to investigate the changes in FC between the two groups. Furthermore, we evaluated the associations between altered thalamus FC and clinical features in AUVP patients using Pearson’s partial correlation.

**Results:**

Compared with HC, AUVP patients showed decreased FC between bilateral thalamus and left insula. We also observed decreased FC between right thalamus and left supramarginal gyrus. Additionally, we found increased FC between left thalamus and right postcentral gyrus (PCG), as well as increased FC between right thalamus and regions of bilateral PCG, right middle frontal gyrus and right middle occipital gyrus in AUVP patients. Furthermore, the FC between left thalamus and left insula was negatively correlated with values of canal paresis in patients with AUVP (*p* = 0.010, *r* = −0.434).

**Conclusion:**

Our results provided first evidence for the decreased thalamo-vestibular cortex pathway, as well as increased thalamo-somatosensory and thalamo-visual cortex pathway in AUVP patients. These findings help us better understand the underlying mechanisms of central dynamic compensatory following an acute unilateral peripheral vestibular damage.

## Introduction

Acute unilateral vestibulopathy (AUVP), also known as vestibular neuritis (VN), is characterized by acute or sub-acute episodes of moderate-to-severe rotational or non-rotational vertigo accompanied by instability and spontaneous horizontal nystagmus (or torsion) without hearing impairment or brainstem dysfunction (Strupp and Magnusson, 2015; Johns and Quinn, 2020; Strupp et al., 2022). AUVP is the second leading cause of peripheral vestibular vertigo (Strupp and Brandt, 2013). The incidence of AUVP ranges from 3.5∼15.5/100,000 (Strupp and Brandt, 2009). AUVP accounts for 3.2%–9% of patients in vertigo or neurology clinics (Strupp and Brandt, 2009). About 2%–11% of AUVP patients relapse, and 10%–15% of AUVP patients have secondary benign paroxysmal positional vertigo (Neuhauser, 2016; Le et al., 2019).

Most AUVP patients experience a quick recovery that seems to be related to a compensatory adaptation for the central vestibular information processing. Previous studies have shown that 30%∼50% of AUVP patients developed chronic dizziness, including persistent postural-perceptual dizziness (Yan et al., 2017; Popkirov et al., 2018). Recent studies suggested that the development of such chronic symptoms following AUVP was predominantly mediated by central mechanisms (inadequate central vestibular compensation) (Best et al., 2006; Panichi et al., 2017). It was reported that the vestibular nucleus might play a crucial role in vestibular compensation in patients with unilateral peripheral vestibular damage or in rats model of acute unilateral labyrinthotomy (Helmchen et al., 2011; Zwergal et al., 2016). A previous positron emission tomography (PET) study has reported changes in regional cerebral glucose metabolism in vestibular cortex in patients with AUVP during acute stage (Bense et al., 2004). Neuroimaging studies using method of voxel-based morphometry have reported gray matter volume changes in regions of vestibular cortex in patients with AUVP (Helmchen et al., 2009; Hong et al., 2014). Functional and structural changes of vestibular cortex in AUVP patients indicate that the vestibular cortex probably plays an important role in vestibular compensation. In the vestibular pathway, peripheral vestibular information is projected through vestibular nucleus to ocular motor nuclei, and in parallel to ascend via thalamus to the central vestibular cortex (Hitier et al., 2014; Kirsch et al., 2016). So far, few studies have focused on the functional compensation of thalamus in patients with AUVP (Becker-Bense et al., 2014; Helmchen et al., 2014; Klingner et al., 2014; Roberts et al., 2018; Cheng et al., 2023). It is currently not clear whether patients with AUVP have abnormal resting-state functional connectivity (FC) of the thalamus.

Therefore, this study aimed to explore the changes in FC focused on bilateral thalamus in patients with AUVP during acute phase using resting-state functional magnetic resonance imaging (fMRI), and to determine whether these FC abnormalities correlate with certain clinical features of the patients. We hypothesized that the process of central vestibular compensation in AUVP patients involved functional changes in the thalamus. It has been suggested that adaptation is one of the strategies for vestibular compensation (Hillier and McDonnell, 2011). The main strategy of adaptation is sensory substitution. Vision and proprioception are the main sources of sensory substitution (Hillier and McDonnell, 2011; Lacour and Bernard-Demanze, 2015). It is believed that thalamus is functionally connected with visual, somatosensory, and vestibular cortex (Wijesinghe et al., 2015; Roy et al., 2022). Thus, we speculated that patients with AUVP might have altered resting-state FC between thalamus and a set of brain regions involved in vestibular, visual, and somatosensory information processing.

## Materials and methods

### Participants

Based on the diagnostic criteria of AUVP/VN from the consensus document of the committee for the classification of vestibular disorders of the Bárány Society (Strupp et al., 2022), 40 right-handed AUVP patients were recruited at the Department of Neurology of the Second Affiliated Hospital of Xuzhou Medical University. All patients were evaluated within 1 week of onset. Each patient underwent a detailed neurological and neuro-otological evaluation, including demographic information, history of present illness, past medical history, neurological and vertigo bedside examination, videonystagmograph, video head impulse test (vHIT), vestibular evoked myogenic potentials (VEMPs, including ocular and cervical VEMPs), rotatory chair test, pure tone test, and measurement of acoustic immittance. In the videonystagmograph evaluation, a patient with AUVP usually characteristically presents as a spontaneous nystagmus (horizontal-torsional, direction-fixed, and enhanced by removal of visual fixation) with a trajectory appropriate to the semicircular canal afferents involved. In the vHIT and rotatory chair test, a patient with AUVP usually shows reduced vestibulo-ocular reflex (VOR) function on the side opposite the direction of the fast phase of the spontaneous nystagmus (VOR gain less than 0.7 with saccades for vHIT, or canal paresis more than 25% for rotatory chair test). In addition, examinations of VEMPs and vHIT help us to differentiate the distribution of nerve injury in AUVP patients. All patients underwent acute or subacute onset of vertigo lasting more than 24 h. No patient had positive neurological signs beyond those of a unilateral peripheral vestibular lesion. None of the patients had hearing impairment or tinnitus, or other otologic symptoms such as otalgia. No patient had previous history of neurological, neuro-otological, psychiatric, or systemic disease. To control for laterality, all patients suffered damage to the left side. Patients with drug or alcohol abuse were excluded. Patients with recurrent AUVP were excluded. Patients who received vestibular function inhibitors within 2 days prior to assessments of vestibular function and fMRI were excluded. For enrolled patients, resting-state fMRI were performed within 1 week of onset, and all fMRI scans were obtained on the same day as the completion of the vestibular function evaluation.

Thirty-five age, sex, and education-matched healthy controls (HC) were recruited. They received resting-state fMRI scanning. They are all right-handed, with no history of vertigo, drug, or alcohol abuse. HC with neurological, neuro-otological, mental, or systemic disorders were excluded. Both patients with AUVP and HC underwent routine MRI (T1WI + T2WI + DWI + FLAIR) scans to rule out occult central nervous system diseases. In addition, subjects with moderate-severe white matter hyperintensity on FLAIR images were excluded. In addition, all participants underwent assessments of Hamilton Anxiety Scale (HAMA), Hamilton Depression Scale (HAMD), and Montreal Cognitive Assessment (MoCA). All scales and neuropsychological tests were completed by a qualified neuropsychological evaluator who was not aware of the grouping of the subjects.

### Imaging acquisition

All MRI images were collected using a 3.0T GE MRI scanner (GE Medical Systems) at the Second Affiliated Hospital of Xuzhou Medical University, including high resolution T1-weighted images (T1WI) and resting-state fMRI. All participants kept their eyes closed and lay still during the MRI scanning. In addition, all subjects were asked not to think about anything during the MRI scanning. The T1WI images were obtained using a 3D-BRAVO sequence with the following parameters: repetition time (TR) = 2,500 ms, echo time (TE) = 3.5 ms, flip angle (FA) = 8°, matrix = 256 × 256, thickness = 1 mm, 156 slices. A fast field echo-planar imaging (EPI) sequence was used to acquire the whole brain fMRI images, the scanning parameters were as follows: TR = 2,000 ms, TE = 30 ms, FA = 90°, field of view (FOV) = 200 × 200 mm, matrix = 64 × 64, thickness/gap = 3.6/0 mm, 210 time points, total scan time = 7 min.

### Imaging preprocessing

For the acquired 210 functional time points, we removed the first 10 time points. The remaining 200 time points were preprocessed by Statistical Parametric Mapping 12 (SPM12)^1^ and CONN toolbox (Version 18b)^2^ working on MATLAB R2023a (MathWorks, Inc., Natick, MA, USA). The preprocessing procedures were the default parameters within the CONN toolbox: (a) functional slice-timing correction was used to reduce the difference in the acquisition time of each layer; (b) functional realignment was adopted to correct head movement within sequence; (c) we performed functional outlier detection with head motion threshold set at 3 mm and global signal threshold set at *z* = 9; (d) in order to narrow the difference in structural center caused by manual positioning, we performed the step of structural center to (0, 0, 0) coordinates (translation); (e) functional segmentation and normalization (DARTEL); (f) spatial smoothing based on a Gaussian kernel of 6-mm full-width at half maximum; (g) band-pass filtering (0.01–0.08 Hz); and (h) nuisance variable regression: including the signals of white matter, cerebrospinal fluid, and the 24 motion parameters. Six AUVP patients and four HC were excluded due to large head motion or poor normalization. Finally, 34 AUVP patients and 31 HC were included in the following analysis.

### Seed-based functional connectivity

Functional connectivity between the selected seeds and the rest of the brain regions was computed with the seed-to-voxel method. The bilateral thalamus were extracted from the FSL Harvard–Oxford atlas. For each seed, we extracted the time-series from the preprocessed functional images. We calculated the Pearson correlation coefficients (*r*) between the seed and all other voxels, which was then converted to *z* scores using Fisher’s r-to-z transformation.

### Statistical analysis

#### Analysis of demography and clinical characteristics

Statistical analyses of demography and clinical characteristics were performed using the Statistical Package for the Social Sciences (SPSS, v22.0) for Windows (SPSS Institute Inc., Chicago, IL, USA). Two-sample *t*-tests were adopted to analyze the differences in age, educational years, scores of HAMA, HAMD, and MoCA, the Chi-square test was used to examine the difference in gender. A *p* < 0.05 was considered significant for all analyses.

#### Analysis of differences in FC

Two-sample *t*-tests were applied to analyze the differences of FC between AUVP patients and HC. Age, gender, education level, scores of MoCA, HAMA, and HAMD were included as covariates. Significance was determined at voxel-level threshold (*p* < 0.001) and cluster-level threshold [*p* < 0.05, false discovery rate (FDR) corrected, two-tailed].

### Correlation analysis

For FC showing significant differences between the two groups, *z*-values were calculated. We performed Pearson’s partial correlation analysis to evaluate the correlations between *z*-values and patients’ clinical features [including duration of AUVP, values of canal paresis (CP), slow-phase velocity of spontaneous nystagmus, scores of VVAS and DHI], controlling for age, gender, education level, scores of MoCA, HAMA, and HAMD. A *p* < 0.05 was considered significant.

## Results

### Demographic and clinical features

The general data of all subjects were summarized in Table 1. There was no significant difference in age, gender, education level, scores of HAMA, HAMD, and MoCA between the two groups (all *p* > 0.05, Table 1). The clinical characteristics of AUVP patients were shown in Table 2. The AUVP patients in the present study suffered from moderate to severe vertigo symptoms according to the scores of VVAS (6.4 ± 1.3), which resulted in severe handicap as identified by scores of DHI (61.1 ± 20.6). Of all the 34 patients with AUVP we enrolled, 10 (29.4%) patients had a clear history of upper respiratory infection before onset. All patients (34, 100%) had left peripherals vestibular lesions. All patients (34, 100%) showed spontaneous nystagmus (Spn), the slow-phase velocity of Spn was 12.1 ± 11.4°/s. Thirty-two (94.1%) patients showed right side Spn and 1 (5.9%) patient showed left side Spn. All patients (34, 100%) showed canal paresis (CP, 60.6% ± 20.0%) during rotatory chair testing. Thirty (88.2%) patients displayed abnormal vHIT (left side VOR gain reduction and saccade waves). Ocular VEMP (oVEMP) was abnormal in 30 cases (88.2%) and cervical VEMP (cVEMP) was abnormal in 13 cases (38.2%). Results of vHIT and VEMPs showed that 20 (58.8%) patients had superior vestibular nerve damage only, 3 (8.9%) patients had inferior vestibular nerve damage only, 1 (2.9%) patient had ampullary nerve damage and 10 (29.4%) patients had whole vestibular nerve damage (Table 3).

**TABLE 1:** General information of the participants.

	AUVP (*n* = 34)	HC (*n* = 31)	*p*-Value
Age (years)	51.3 ± 10.9	48.7 ± 13.4	0.108
Gender (male/female)	18/16	14/17	0.705
Education (years)	14.3 ± 3.6	15.1 ± 3.6	0.183
HAMA scores	19.4 ± 12.5	16.3 ± 11.1	0.091
HAMD scores	12.8 ± 7.6	9.7 ± 5.3	0.084
MoCA scores	28.4 ± 1.2	28.1 ± 1.3	0.477

AUVP, acute unilateral vestibulopathy; HC, healthy control; HAMA, Hamilton Anxiety Scale; HAMD, Hamilton Depression Scale; MoCA, Montreal Cognitive Assessment Scale.

**TABLE 2:** Clinical features of the AUVP patients (*n* = 34).

VVAS scores	6.4 ± 1.3
DHI scores	61.1 ± 20.6
Duration of AUVP (days)	4.5 ± 2.2
History of pre-onset URI (*n*, %)	10 (29.4%)
CP (+) (*n*, %)	34 (100%)
CP values (%)	60.6% ± 20.0%
vHIT (+) [L (*n*), %]	30 (88.2%)
oVEMP (+) (*n*, %)	30 (88.2%)
cVEMP (+) (*n*, %)	13 (38.2%)
Spn [R (*n*), %]	32 (94.1%)
Spn-SPV (°/s)	12.1 ± 11.4°/s

AUVP, acute unilateral vestibulopathy; VVAS, Vertigo Visual Analog Scale; DHI, dizziness handicap inventory; URI, upper respiratory infection; L, left; R, right; CP, canal paresis; vHIT, video head impulse test; oVEMP, ocular vestibular evoked myogenic potentials; cVEMP, cervical vestibular evoked myogenic potentials; Spn, spontaneous nystagmus; SPV, slow-phase velocity; +, abnormal.

**TABLE 3:** Distribution characteristics of nerve injury in patients with AUVP.

Involved VN	Case number (%)	vHIT-H	vHIT-A	vHIT-P	oVEMP	cVEMP
Superior VN	20 (58.8%)	+	+	−	+	−
Whole VN	10 (29.4%)	+	+	+	+	+
Inferior VN	3 (8.9%)	−	−	+	−	+
Ampullary nerve	1 (2.9%)	+	+	−	−	−

AUVP, acute unilateral vestibulopathy; VN, vestibular nerve; vHIT, video head impulse test; H, horizontal semicircular canal; A, anterior semicircular canal; P, posterior semicircular canal; oVEMP, ocular vestibular evoked myogenic potentials; cVEMP, cervical vestibular evoked myogenic potentials; +, abnormal;−, normal.

### FC differences between groups

**FIGURE 1:**
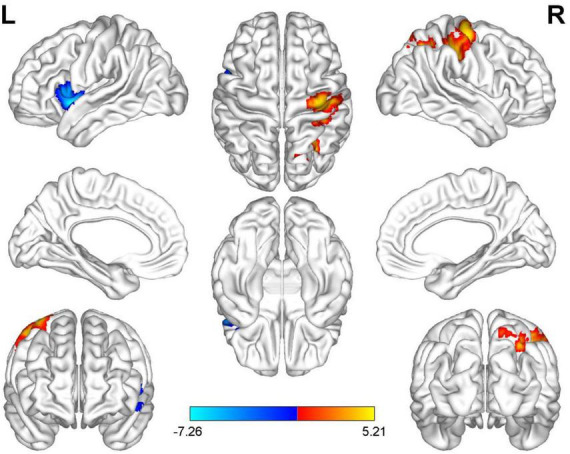
Differences in functional connectivity (FC) between patients with AUVP and healthy controls when the left thalamus was chosen as a seed. Blue and cyan regions suggest decreased FC, while red and yellow areas indicate increased FC [voxel-level threshold (*p* < 0.001) and cluster-level threshold (two-tailed, *p* < 0.05, FDR correction)]. AUVP, acute unilateral vestibulopathy; FDR, false discovery rate; L, left; R, right.

**FIGURE 2:**
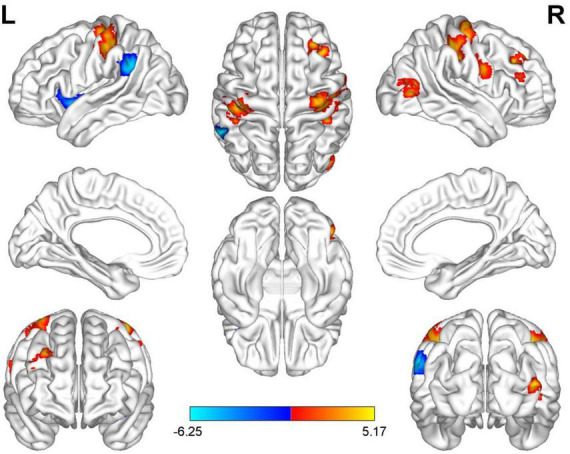
Differences in functional connectivity (FC) between patients with AUVP and healthy controls when the right thalamus was chosen as a seed. Blue and cyan regions suggest decreased FC, while red and yellow areas indicate increased FC [voxel-level threshold (*p* < 0.001) and cluster-level threshold (two-tailed, *p* < 0.05, FDR correction)]. AUVP, acute unilateral vestibulopathy; FDR, false discovery rate; L, left; R, right.

Compared with HC, AUVP patients showed increased FC between the left thalamus and the right postcentral gyrus (PCG) (Table 4 and Figure 1). We also observed decreased FC between the left thalamus and the left insula in patients with AUVP compared with HC (Table 4 and Figure 1). In addition, compared with HC, AUVP patients showed increased FC between the right thalamus and brain regions of the bilateral PCG, right middle frontal gyrus (MFG) and right middle occipital gyrus (MOG) (Table 5 and Figure 2). We also found decreased FC between the right thalamus and brain regions of the left insula and the left supramarginal gyrus (SMG) in AUVP patients (Table 5 and Figure 2).

**TABLE 4:** Altered functional connectivity of the left thalamus in AUVP patients compared with HC.

Brain regions	Voxel size	Peak MNI coordinates *x*, *y*, *z*	Peak *t*-score	AAL	BA
R PCG	440	39, −27, 48	5.2105	Postcentral_R	1
L insula	378	−39, 9, −6	−7.2593	Insula_L	13

The results were assigned thresholds at voxel-level threshold (*p* < 0.001) and cluster-level threshold (two-tailed, *p* < 0.05) with false discovery rate (FDR) correction. AUVP, acute unilateral vestibulopathy; HC, healthy control; MNI, Montreal Neurological Institute; AAL, anatomical automatic labeling; BA, Brodmann area; L, left; R, right; PCG, postcentral gyrus.

**TABLE 5:** Altered functional connectivity of the right thalamus in AUVP patients compared with HC.

Brain regions	Voxel size	Peak MNI coordinates *x*, *y*, *z*	Peak *t*-score	AAL	BA
R PCG	328	48, −21, 36	5.1682	Postcentral_R	1
L PCG	177	−45, −21, 63	4.6665	Postcentral_L	4
R MFG	75	30, 39, 42	4.0589	Frontal_Mid_R	9
R MOG	66	45, −78, 12	4.6553	Occipital_Mid_R	19
L insula	112	−39, 3, −6	−5.9203	Insula_L	13
L SMG	90	−59, −47, 31	−6.2496	SupraMarginal_L	39

The results were assigned thresholds at a voxel-level threshold (*p* < 0.001) and a cluster-level threshold (two-tailed, *p* < 0.05) with false discovery rate (FDR) correction. AUVP, acute unilateral vestibulopathy; HC, healthy control; MNI, Montreal Neurological Institute; AAL, anatomical automatic labeling; BA, Brodmann area; L, left; R, right; PCG, postcentral gyrus; MFG, middle frontal gyrus; MOG, middle occipital gyrus; SMG, supramarginal gyrus.

### Correlation results

**FIGURE 3:**
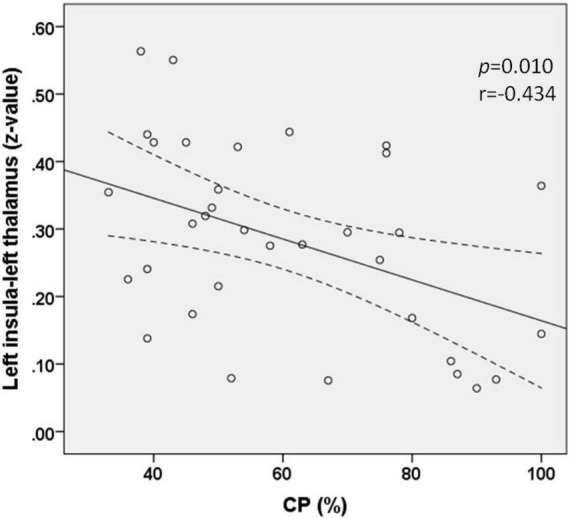
Functional connectivity between the left insula and left thalamus was negatively correlated with the value of canal paresis (CP) in patients with acute unilateral vestibulopathy (*p* = 0.010, *r* = –0.434).

Correlation analysis revealed that the FC (*z*-value) between the left thalamus and the left insula was negatively correlated with the value of CP in patients with AUVP (*p* = 0.010, *r* = −0.434; Figure 3). There was no significant correlation between the FC (*z*-value) and patients’ clinical features of disease duration, slow-phase velocity of Spn, and scores of VVAS and DHI (all *p* > 0.05).

## Discussion

To our knowledge, the present study is the only one which explored resting-state FC changes focusing on thalamus in patients with AUVP. We found that AUVP patients showed abnormal thalamus resting-state FC. In addition, our results suggested that the altered thalamus FC was correlated with the severity of peripheral vestibular impairment (CP values) in the AUVP patients.

The thalamus is believed to receive vestibular input from vestibular nuclei and cerebellum, and transmit vestibular input to the central vestibular cortex (Hitier et al., 2014; Kirsch et al., 2016). Previous studies have suggested that the thalamus was involved in the processing of vestibular information (Asanuma et al., 1983; Indovina et al., 2005). Functional and structural abnormalities of thalamus have been reported in vestibular diseases. For example, Meniere’s disease, vestibular migraine and visually induced dizziness (Van Ombergen et al., 2017; Borghammer and Ovesen, 2020; Chen et al., 2022; Zhe et al., 2023). In patients with AUVP, altered regional cerebral glucose metabolism in the thalamus was previously reported during the acute stage (Bense et al., 2004), which was partially similar to the results of the present study. Our results of the altered thalamus FC supported our hypothesis and potentially suggested that changes in thalamus functional activity were involved in the process of vestibular compensation in AUVP patients.

The insula is an important component of parieto-insular vestibular cortex (PIVC) (Eickhoff et al., 2006; Frank and Greenlee, 2018). The PIVC is mainly located in the posterior insula, retroinsular region and the parietal operculum, and is now generally considered to be the primary vestibular cortex in humans (Eickhoff et al., 2006; Frank and Greenlee, 2018). The insula receives vestibular inputs from thalamus and plays a considerable role in vestibulo-thalamo-PIVC pathway (Lopez et al., 2012; zu Eulenburg et al., 2012). An 18F-fluorodeoxyglucose PET study reported increased regional cerebral glucose metabolism in posterior insula in AUVP patients (Bense et al., 2004). Studies using voxel-based morphometry analysis found increased gray matter volume in the insula in AUVP patients compared with HC, and in AUVP patients at the restoration stage (3 months) compared with the same patients at the acute stage (onset within 72 h) (Helmchen et al., 2009; Hong et al., 2014). Recently, a neuroimaging study reported that AUVP patients at acute stage showed decreased FC between left lingual gyrus and right insula, indicating decreased FC between visual and vestibular cortex in patients with AUVP (Cheng et al., 2023). Partially consistent with previous functional and structural neuroimaging studies (Bense et al., 2004; Helmchen et al., 2009; Hong et al., 2014; Cheng et al., 2023), the present study found that AUVP patients with left peripherals vestibular lesions showed decreased FC between the left insula and bilateral thalamus, indicating decreased thalamo-vestibular cortex neural pathway. In addition, our AUVP patients showed a significant negative correlation between the increased CP (the greater the CP value, the more serious the damage of vestibular function) and the decreased FC of left thalamus-insula. The decrease in resting-state FC between the left thalamus and left insula was probably related to the impaired left side peripherals vestibular function in the AUVP patients with left peripherals vestibular lesions.

This study also observed decreased FC between the right thalamus and left SMG in AUVP patients with left peripherals vestibular lesions. The SMG within the inferior parietal lobule (IPL) has been shown to be activated by vestibular stimulation (Lopez and Blanke, 2011). It was also reported that a left parietal lobe infarction involving the SMG presented as isolated positional vertigo with rightward horizontal nystagmus (Naganuma et al., 2006). The SMG is a key region identified in previous studies for vestibular information processing (Lopez and Blanke, 2011; zu Eulenburg et al., 2012; Dieterich and Brandt, 2018). The functional and structural changes in SMG have been demonstrated in vestibular disorders like benign paroxysmal positional vertigo with residual dizziness, persistent postural-perceptual dizziness, and vestibular migraine (Zhe et al., 2020; Lin et al., 2023; Maywald et al., 2023). A recent fMRI study has revealed decreased FC between the left lingual gyrus and the bilateral SMG in patients with AUVP (Cheng et al., 2023). The current study found decreased FC between the right thalamus and left SMG. This result also indicated decreased thalamo-vestibular cortex neural pathway in AUVP patients.

The PCG is part of the parietal lobe and is known as primary somatosensory cortex of human beings (Kaas et al., 1979; Geyer et al., 2000). In AUVP patients, increased regional homogeneity (ReHo) values in the right PCG and decreased regional cerebral glucose metabolism in PCG were previously reported by resting-state fMRI and 18F-fluorodeoxyglucose PET studies (Bense et al., 2004; Cheng et al., 2023). The present study observed increased FC between the left thalamus and right PCG, and between the right thalamus and bilateral PCG. These results suggested increased thalamo-somatosensory cortex neural pathway in AUVP patients. We also discovered increased FC between the right thalamus and right MFG in patients with AUVP. The MFG is a crucial component of the prefrontal cortex, which is reported to be involved in the attention network (Vossel et al., 2014). It was reported that the MFG was activated in response to an active unilateral knee joint position sense test, suggesting that the MFG was involved in the processing of proprioception (Strong et al., 2023). Another study suggested that the MFG might play a role in the integration of proprioceptive information in the process of sensory and motor information integration (Stock et al., 2013). Therefore, we speculated that the increased FC between the right thalamus and right MFG might also reflect increased thalamo-somatosensory cortex neural pathway in AUVP patients. Similar to our results, it was reported that there was a shift to the somatosensory system due to an elevated processing of proprioceptive information in the right gracile nucleus in AUVP patients during chronic stage (zu Eulenburg et al., 2010). However, similar to most previous studies (zu Eulenburg et al., 2010; Hong et al., 2014; Klingner et al., 2014; Roberts et al., 2018; Cheng et al., 2023), we did not evaluate the vestibulospinal reflex (VSR) pathway for the AUVP patients as the VSR pathway is involved in acute vertigo and runs through the thalamus (Helmchen et al., 2014; Carmona et al., 2023). Therefore, the increased thalamo-somatosensory cortex neural pathway observed in AUVP patients probably had something to do with the deficit in VSR pathway.

In addition, the current study found increased FC between the right thalamus and right MOG in AUVP patients. The MOG is known to be an important part of the lateral visual network and belongs to the secondary visual cortex. Thus, our study indicated increased thalamo-visual cortex neural pathway in AUVP patients. Previous neuroimaging studies have also demonstrated the altered function of visual cortex in AUVP patients (Bense et al., 2004; Cheng et al., 2023) and in patients with chronic unilateral vestibulopathy (CUVP) (Si et al., 2022). In addition, a voxel-based morphometry study observed increased gray matter volume in the MOG in AUVP patients with disease duration longer than 3 months compared with the same patients at acute stage (onset within 72 h) (Cheng et al., 2023).

It is well known that the maintenance of human postural balance depends on the close cooperation among vestibular, visual, and somatosensory systems. We believed that the interaction and integration among different senses makes it possible for sensory substitution in AUVP patients. When vestibular sense is absent, AUVP patients may compensate for the missing vestibular sense with visual and somatosensory senses to maintain postural balance, visual stability and perception of spatial location. The present study observed decreased thalamo-vestibular cortex neural pathway, as well as increased thalamo-somatosensory and thalamo-visual cortex neural pathway in AUVP patients during acute phase. This potentially indicated a powerful functional central vestibular compensation within the visual and somatosensory systems when the peripheral vestibular system undergoes a substantial loss of function. Our findings of functional vestibular compensation echoed an earlier voxel-based morphometry research that provided evidence for the volumetric increases in the visual and somatosensory cortices from the perspective of structural vestibular compensation in AUVP patients during chronic phase (zu Eulenburg et al., 2010).

### Limitations

This study had several potential limitations. First, the sample size was relatively small and the data were gathered from a single center. Second, the seeds (bilateral thalamus) we adopted contained the whole thalamus. In fact, the thalamus can be divided into several sub-regions, in order to improve the specificity of the analysis, future study should define anatomical parcellation of the thalamus more specifically. The third potential limitation was that we only adopted method of seed-based FC, future studies should combine other methods to further explore the neuroimaging changes of AUVP, for example, independent component analysis (ICA), functional network connectivity (FNC), dynamic FNC, VBM, and so on. Fourth, we did not evaluate the VSR pathway for the AUVP patients. Finally, we did not follow up patients with AUVP to observe changes in brain functional activity after 3 months to further verify the results of this study.

## Conclusion

In conclusion, our results provided first evidence for the decreased thalamo-vestibular cortex neural pathway, as well as increased thalamo-somatosensory and thalamo-visual cortex neural pathway in AUVP patients during acute phase. These findings help us better understand the underlying mechanisms of central dynamic compensatory following an acute unilateral peripheral vestibular damage.

## Data availability statement

The original contributions presented in this study are included in the article/supplementary material, further inquiries can be directed to the corresponding authors.

## Ethics statement

The studies involving humans were approved by the Ethics Committee of the Second Affiliated Hospital of Xuzhou Medical University. The studies were conducted in accordance with the local legislation and institutional requirements. The participants provided their written informed consent to participate in this study.

## Author contributions

ZC: Conceptualization, Data curation, Formal analysis, Funding acquisition, Investigation, Methodology, Project administration, Software, Visualization, Writing – original draft, Writing – review & editing. YC: Formal analysis, Investigation, Visualization, Writing – original draft, Writing – review & editing. YL: Investigation, Methodology, Visualization, Writing – review & editing. HL: Formal analysis, Investigation, Methodology, Writing – review & editing. X-EW: Formal analysis, Investigation, Visualization, Writing – review & editing. CL: Formal analysis, Investigation, Writing – review & editing. DL: Investigation, Visualization, Writing – review & editing. LX: Data curation, Investigation, Project administration, Supervision, Writing – review & editing. LR: Formal analysis, Investigation, Project administration, Software, Supervision, Writing – review & editing.

## Footnotes

1
https://www.fil.ion.ucl.ac.uk/spm/software/spm12

2
http://www.nitrc.org/projects/conn

## References

[B1] Asanuma C. Thach W. T. Jones E. G. (1983). Distribution of cerebellar terminations and their relation to other afferent terminations in the ventral lateral thalamic region of the monkey. *Brain Res.* 286 237–265. 10.1016/0165-0173(83)90015-2 6189561

[B2] Becker-Bense S. Dieterich M. Buchholz H. G. Bartenstein P. Schreckenberger M. Brandt T. (2014). The differential effects of acute right- vs. left-sided vestibular failure on brain metabolism. *Brain Struct. Funct.* 219 1355–1367. 10.1007/s00429-013-0573-z 23686397

[B3] Bense S. Bartenstein P. Lochmann M. Schlindwein P. Brandt T. Dieterich M. (2004). Metabolic changes in vestibular and visual cortices in acute vestibular neuritis. *Ann. Neurol.* 56 624–630. 10.1002/ana.20244 15449325

[B4] Best C. Eckhardt-Henn A. Diener G. Bense S. Breuer P. Dieterich M. (2006). Interaction of somatoform and vestibular disorders. *J. Neurol. Neurosurg. Psychiatry* 77 658–664. 10.1136/jnnp.2005.072934 16614028 PMC2117464

[B5] Borghammer P. Ovesen T. (2020). PET visualized stimulation of the vestibular organ in Menière’s disease. *Front. Neurol.* 11:11. 10.3389/fneur.2020.00011 32047473 PMC6997538

[B6] Carmona S. Martínez C. Zalazar G. Koohi N. Kaski D. (2023). Acute truncal ataxia without nystagmus in patients with acute vertigo. *Eur. J. Neurol.* 30 1785–1790. 10.1111/ene.15729 36752029

[B7] Chen Z. Xiao L. Liu H. Zhang Q. Wang Q. Lv Y. (2022). Altered thalamo-cortical functional connectivity in patients with vestibular migraine: A resting-state fMRI study. *Neuroradiology* 64 119–127. 10.1007/s00234-021-02777-w 34374821

[B8] Cheng Q. Ren A. Han Y. Jin X. Pylypenko D. Yu D. (2023). Assessment of functional and structural brain abnormalities with resting-state functional MRI in patients with vestibular neuronitis. *Acta Radiol.* 64 3024–3031. 10.1177/02841851231203569 37807650

[B9] Dieterich M. Brandt T. (2018). The parietal lobe and the vestibular system. *Handb. Clin. Neurol.* 151 119–140. 10.1016/B978-0-444-63622-5.00006-1 29519455

[B10] Eickhoff S. B. Weiss P. H. Amunts K. Fink G. R. Zilles K. (2006). Identifying human parieto-insular vestibular cortex using fMRI and cytoarchitectonic mapping. *Hum. Brain Mapp.* 27 611–621. 10.1002/hbm.20205 16281284 PMC6871353

[B11] Frank S. M. Greenlee M. W. (2018). The parieto-insular vestibular cortex in humans: More than a single area? *J. Neurophysiol.* 120 1438–1450. 10.1152/jn.00907.2017 29995604

[B12] Geyer S. Schormann T. Mohlberg H. Zilles K. (2000). Areas 3a, 3b, and 1 of human primary somatosensory cortex. Part 2. Spatial normalization to standard anatomical space. *Neuroimage* 11 684–696. 10.1006/nimg.2000.0548 10860796

[B13] Helmchen C. Klinkenstein J. Krüger A. Gliemroth J. Mohr C. Sander T. (2011). Structural brain changes following peripheral vestibulo-cochlear lesion may indicate multisensory compensation. *J. Neurol. Neurosurg. Psychiatry* 82 309–316. 10.1136/jnnp.2010.204925 20802221

[B14] Helmchen C. Klinkenstein J. Machner B. Rambold H. Mohr C. Sander T. (2009). Structural changes in the human brain following vestibular neuritis indicate central vestibular compensation. *Ann. N. Y. Acad. Sci.* 1164 104–115. 10.1111/j.1749-6632.2008.03745.x 19645887

[B15] Helmchen C. Ye Z. Sprenger A. Münte T. F. (2014). Changes in resting-state fMRI in vestibular neuritis. *Brain Struct. Funct.* 219 1889–1900. 10.1007/s00429-013-0608-5 23881293

[B16] Hillier S. L. McDonnell M. (2011). Vestibular rehabilitation for unilateral peripheral vestibular dysfunction. *Clin. Otolaryngol.* 36 248–249. 10.1111/j.1749-4486.2011.02309.x 21752206

[B17] Hitier M. Besnard S. Smith P. F. (2014). Vestibular pathways involved in cognition. *Front. Integr. Neurosci.* 8:59. 10.3389/fnint.2014.00059 25100954 PMC4107830

[B18] Hong S. K. Kim J. H. Kim H. J. Lee H. J. (2014). Changes in the gray matter volume during compensation after vestibular neuritis: A longitudinal VBM study. *Restor. Neurol. Neurosci.* 32 663–673. 10.3233/RNN-140405 25096973

[B19] Indovina I. Maffei V. Bosco G. Zago M. Macaluso E. Lacquaniti F. (2005). Representation of visual gravitational motion in the human vestibular cortex. *Science* 308 416–419. 10.1126/science.1107961 15831760

[B20] Johns P. Quinn J. (2020). Clinical diagnosis of benign paroxysmal positional vertigo and vestibular neuritis. *CMAJ* 192 182–186. 10.1503/cmaj.190334 32094268 PMC7043823

[B21] Kaas J. H. Randall J. N. Sur M. Lin C. S. Merzenich M. M. (1979). Multiple representations of the body within the primary somatosensory cortex of primates. *Science* 204 521–523. 10.1126/science.107591 107591

[B22] Kirsch V. Keeser D. Hergenroeder T. Erat O. Ertl-Wagner B. Brandt T. (2016). Structural and functional connectivity mapping of the vestibular circuitry from human brainstem to cortex. *Brain Struct. Funct.* 221 1291–1308. 10.1007/s00429-014-0971-x 25552315

[B23] Klingner C. M. Volk G. F. Brodoehl S. Witte O. W. Guntinas-Lichius O. (2014). Disrupted functional connectivity of the default mode network due to acute vestibular deficit. *Neuroimage Clin.* 6 109–114. 10.1016/j.nicl.2014.08.022 25379422 PMC4215422

[B24] Lacour M. Bernard-Demanze L. (2015). Interaction between vestibular compensation mechanisms and vestibular rehabilitation therapy: 10 recommendations for optimal functional recovery. *Front. Neurol.* 5:285. 10.3389/fneur.2014.00285 25610424 PMC4285093

[B25] Le T. N. Westerberg B. D. Lea J. (2019). Vestibular neuritis: Recent advances in etiology, diagnostic evaluation, and treatment. *Adv. Otorhinolaryngol.* 82 87–92. 10.1159/000490275 30947184

[B26] Lin C. Liu D. Liu Y. Chen Z. Wei X. Liu H. (2023). Altered functional activity of the precuneus and superior temporal gyrus in patients with residual dizziness caused by benign paroxysmal positional vertigo. *Front. Neurosci.* 17:1221579. 10.3389/fnins.2023.1221579 37901419 PMC10600499

[B27] Lopez C. Blanke O. (2011). The thalamocortical vestibular system in animals and humans. *Brain Res. Rev.* 67 119–146. 10.1016/j.brainresrev.2010.12.002 21223979

[B28] Lopez C. Blanke O. Mast F. W. (2012). The human vestibular cortex revealed by coordinate-based activation likelihood estimation meta-analysis. *Neuroscience* 212 159–179. 10.1016/j.neuroscience.2012.03.028 22516007

[B29] Maywald M. Pogarell O. Levai S. Paolini M. Tschentscher N. Rauchmann B. S. (2023). Neurofunctional differences and similarities between persistent postural-perceptual dizziness and anxiety disorder. *Neuroimage Clin.* 37:103330. 10.1016/j.nicl.2023.103330 36696807 PMC9879992

[B30] Naganuma M. Inatomi Y. Yonehara T. Fujioka S. Hashimoto Y. Hirano T. (2006). Rotational vertigo associated with parietal cortical infarction. *J. Neurol. Sci.* 246 159–161. 10.1016/j.jns.2006.02.012 16563439

[B31] Neuhauser H. K. (2016). The epidemiology of dizziness and vertigo. *Handb. Clin. Neurol.* 137 67–82. 10.1016/B978-0-444-63437-5.00005-4 27638063

[B32] Panichi R. Faralli M. Bruni R. Kiriakarely A. Occhigrossi C. Ferraresi A. (2017). Asymmetric vestibular stimulation reveals persistent disruption of motion perception in unilateral vestibular lesions. *J. Neurophysiol.* 118 2819–2832. 10.1152/jn.00674.2016 28814637 PMC5680356

[B33] Popkirov S. Staab J. P. Stone J. (2018). Persistent postural-perceptual dizziness (PPPD): A common, characteristic and treatable cause of chronic dizziness. *Pract. Neurol.* 18 5–13. 10.1136/practneurol-2017-001809 29208729

[B34] Roberts R. E. Ahmad H. Patel M. Dima D. Ibitoye R. Sharif M. (2018). An fMRI study of visuo-vestibular interactions following vestibular neuritis. *Neuroimage Clin.* 20 1010–1017. 10.1016/j.nicl.2018.10.007 30336357 PMC6197146

[B35] Roy D. S. Zhang Y. Halassa M. M. Feng G. (2022). Thalamic subnetworks as units of function. *Nat. Neurosci.* 25 140–153. 10.1038/s41593-021-00996-1 35102334 PMC9400132

[B36] Si L. Cui B. Li Z. Li X. Li K. Ling X. (2022). Altered resting-state intranetwork and internetwork functional connectivity in patients with chronic unilateral vestibulopathy. *J. Magn. Reson. Imaging* 56 291–300. 10.1002/jmri.28031 34921750 PMC9299943

[B37] Stock A. K. Wascher E. Beste C. (2013). Differential effects of motor efference copies and proprioceptive information on response evaluation processes. *PLoS One* 8:e62335. 10.1371/journal.pone.0062335 23658624 PMC3637248

[B38] Strong A. Grip H. Arumugam A. Boraxbekk C. Selling J. Häger C. K. (2023). Right hemisphere brain lateralization for knee proprioception among right-limb dominant individuals. *Front. Hum. Neurosci.* 17:969101. 10.3389/fnhum.2023.969101 36742357 PMC9892188

[B39] Strupp M. Brandt T. (2009). Vestibular neuritis. *Semin. Neurol.* 29 509–519. 10.1055/s-0029-1241040 19834862

[B40] Strupp M. Brandt T. (2013). Peripheral vestibular disorders. *Curr. Opin. Neurol.* 26 81–89. 10.1097/WCO.0b013e32835c5fd4 23254559

[B41] Strupp M. Magnusson M. (2015). Acute unilateral vestibulopathy. *Neurol. Clin.* 33 669–685. 10.1016/j.ncl.2015.04.012 26231279

[B42] Strupp M. Bisdorff A. Furman J. Hornibrook J. Jahn K. Maire R. (2022). Acute unilateral vestibulopathy/vestibular neuritis: Diagnostic criteria. *J. Vestib. Res.* 32 389–406. 10.3233/VES-220201 35723133 PMC9661346

[B43] Van Ombergen A. Heine L. Jillings S. Roberts R. E. Jeurissen B. Rompaey V. V. (2017). Altered functional brain connectivity in patients with visually induced dizziness. *Neuroimage Clin.* 14 538–545. 10.1016/j.nicl.2017.02.020 28331800 PMC5345975

[B44] Vossel S. Geng J. J. Fink G. R. (2014). Dorsal and ventral attention systems: Distinct neural circuits but collaborative roles. *Neuroscientist* 20 150–159. 10.1177/1073858413494269 23835449 PMC4107817

[B45] Wijesinghe R. Protti D. A. Camp A. J. (2015). Vestibular interactions in the thalamus. *Front. Neural Circuits* 9:79. 10.3389/fncir.2015.00079 26696836 PMC4667082

[B46] Yan Z. Cui L. Yu T. Liang H. Wang Y. Chen C. (2017). Analysis of the characteristics of persistent postural-perceptual dizziness: A clinical-based study in China. *Int. J. Audiol.* 56 33–37. 10.1080/14992027.2016.1211763 27686369

[B47] Zhe X. Gao J. Chen L. Zhang D. Tang M. Yan X. (2020). Altered structure of the vestibular cortex in patients with vestibular migraine. *Brain Behav.* 10:e01572. 10.1002/brb3.1572 32157823 PMC7177586

[B48] Zhe X. Tang M. Ai K. Lei X. Zhang X. Jin C. (2023). Decreased ALFF and functional connectivity of the thalamus in vestibular migraine patients. *Brain Sci.* 13:183. 10.3390/brainsci13020183 36831726 PMC9954115

[B49] zu Eulenburg P. Caspers S. Roski C. Eickhoff S. B. (2012). Meta-analytical definition and functional connectivity of the human vestibular cortex. *Neuroimage* 60 162–169. 10.1016/j.neuroimage.2011.12.032 22209784

[B50] zu Eulenburg P. Stoeter P. Dieterich M. (2010). Voxel-based morphometry depicts central compensation after vestibular neuritis. *Ann. Neurol.* 68 241–249. 10.1002/ana.22063 20695016

[B51] Zwergal A. Schlichtiger J. Xiong G. Beck R. Günther L. Schniepp R. (2016). Sequential [(18)F]FDG μPET whole-brain imaging of central vestibular compensation: A model of deafferentation-induced brain plasticity. *Brain Struct. Funct.* 221 159–170. 10.1007/s00429-014-0899-1 25269833

